# Effect of electrode materials on resistive switching behaviour of NbO_x_-based memristive devices

**DOI:** 10.1038/s41598-023-44110-w

**Published:** 2023-10-09

**Authors:** Giuseppe Leonetti, Matteo Fretto, Fabrizio Candido Pirri, Natascia De Leo, Ilia Valov, Gianluca Milano

**Affiliations:** 1 Department of Applied Science and Technology (DISAT), Politecnico di Torino, C.So Duca Degli Abruzzi 24, 10129 Turin, Italy; 2 Advanced Materials Metrology and Life Sciences Division, Istituto Nazionale Di Ricerca Metrologica (INRiM), Strada Delle Cacce 91, 10135 Turin, Italy; 3 Institute of Electrochemistry and Energy System, Forschungszentrum Jülich, WilhelmJohnen-Straße, 52428 Jülich, Germany; 4 “Acad. Evgeni Budevski” IEE-BAS, Bulgarian Academy of Sciences (BAS), Acad. G. Bonchev Str, Block 10, 1113 Sofia, Bulgaria

**Keywords:** Nanoscale materials, Physics, Applied physics, Electronics, photonics and device physics, Nanoscience and technology, Nanoscale devices, Electronic devices, Materials science, Materials for devices, Electronic devices, Engineering, Electrical and electronic engineering

## Abstract

Memristive devices that rely on redox-based resistive switching mechanism have attracted great attention for the development of next-generation memory and computing architectures. However, a detailed understanding of the relationship between involved materials, interfaces, and device functionalities still represents a challenge. In this work, we analyse the effect of electrode metals on resistive switching functionalities of NbO_x_-based memristive cells. For this purpose, the effect of Au, Pt, Ir, TiN, and Nb top electrodes was investigated in devices based on amorphous NbO_x_ grown by anodic oxidation on a Nb substrate exploited also as counter electrode. It is shown that the choice of the metal electrode regulates electronic transport properties of metal–insulator interfaces, strongly influences the electroforming process, and the following resistive switching characteristics. Results show that the electronic blocking character of Schottky interfaces provided by Au and Pt metal electrodes results in better resistive switching performances. It is shown that Pt represents the best choice for the realization of memristive cells when the NbO_x_ thickness is reduced, making possible the realization of memristive cells characterised by low variability in operating voltages, resistance states and with low device-to-device variability. These results can provide new insights towards a rational design of redox-based memristive cells.

## Introduction

Memristive devices whose functionalities rely on resistive switching (RS) phenomena represent promising candidates for next generation memories as well as for the development of neuromorphic computing architectures^1–6^.The simplest way to realise a memristive device is by sandwiching an insulator material between two metal electrodes, to achieve a so-called metal–insulator-metal (MIM) structure. In these devices, the switching mechanism relies not only on the insulator material but also on the choice of metal electrodes and the metal–insulator interface properties^4,7–10^. In this context, an mportant class of RS devices is represented by the Valence Change Memory (VCM) cells where the insulator material is sandwiched in between an electrochemically inert material where the switching process takes place (i.e., active interface) and a counter material that usually forms an ohmic contact^10^.

As insulator layers, a wide range of transition metal oxides have been considered^9–12^. Among metal oxides, NbO_x_ thin films have recently attracted great attention as insulator layers for the realization of memristive devices^13–33^. Besides resistive switching devices have been realized by depositing NbO_x_ thin films by means of radio frequency (RF) sputtering, Atomic Layer Deposition (ALD), Physical Layer Deposition (PLD), and other Complementary Metal Oxide Semiconductor (CMOS) compatible techniques^16,18,19,26^, anodic oxidation has been recently proposed as an alternative grown technique for the realization of NbO_x_ thin films with resistive switching capabilities^21,22,25,34^. While in these works the resistive switching capabilities have been analysed by considering peculiar metal electrode configurations, a relationship in-between the choice of metal electrodes and resistive switching functionalities in NbO_x_-based devices still have to be established.

In the present work, we systematically investigated the effect of different top electrode material on resistive switching properties of NbO_x_-based memristive devices. For this purpose, the effect of several top electrodes (TEs) materials on NbO_x_ grown by anonic oxidation on a Nb substrate exploited also as counter electrode has been analysed by keeping fixed the Nb counter electrode. Analysed TE materials include high work function metals such as Au, Pt and Ir and a low oxygen affinity material compound such as TiN. As a reference, a symmetrically contacted NbO_x_ with Nb electrodes have been also considered. A detailed analysis of the pristine states of the fabricated cells is reported, analysing how the choice of the TE material influences the electronic transport properties. By analysing the electroforming process and resistive switching functionalities, selection criteria for the choice of the TE materials in NbO_x_ memristive cells are discussed.

## Results and discussion

### Electrode-dependent electronic transport properties in the pristine state

**Figure 1:**
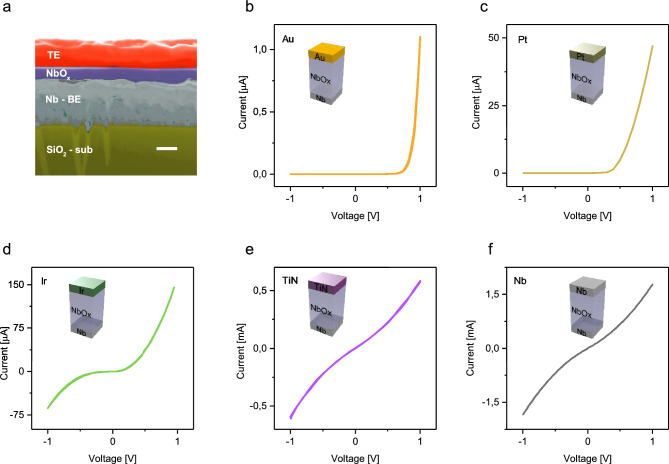
(**a**) Cross-section of a Au/NbO_x_/Nb cell showing the compactness of the anodic grown oxide layer (scale bar is 100 nm, image is in false colours). Typical pristine state curves for the structure NbO_x_/Nb terminated by (**b**) Au, (**c**) Pt, (**d**) Ir, (**e**) TiN, and (**f**) Nb. Measurements are referred to 60 nm NbO_x_ devices contacted by 50 × 50 µm^2^ top electrodes.

Resistive switching devices were fabricated with the typical MIM structure in which a thin layer of anodic NbO_x_ is sandwiched between a common bottom electrode (BE) of Nb and a TE metal chosen among Au, Pt, Ir, TiN, and Nb. An example of the cross-section of the device structure can be found in Fig. 1a in which it can be appreciated the compactness of the NbO_x_ layer and the smoothness of the BE interface due to the anodic oxidation process, as confirmed also by Transmission Electron Microscopy (TEM) analysis reported in our previous work^25^. Note that a detailed chemical and structural analysis revealed that the NbO_x_ is amorphous and is characterised by the presence of Nb(+ 5) oxidation state on the top of the anodized film and Nb(+ 2) oxidation state at the interface with the common Nb BE, as investigated in our previous work^25^. Note that while the same oxidation states are then found after the deposition with the Au TE^25^, chemical properties at the metal/NbO_x_ interface with the TE may locally slightly vary the oxide stoichiometry due to the interaction with the TE metal. To have a comparison between the effect of different TE metals, each type of cell was studied with the same electrical scheme, with the BE grounded and the bias voltage applied to the TE.

The pristine states (i.e., the resistance state before switching events) of memristive cells with different electrodes have been investigated through *I-V* characteristics reported in Fig. 1b–f (log scale plots can be found in Supplementary Figure S1). In this case, the electronic transport mechanism is regulated by metal–insulator interfaces where Schottky barriers are expected. In this context, the MIM structure can be represented as back-to-back Schottky diodes with the NbO_x_ series resistance^35–37^. As expected, results show that the choice of the TE electrode metal strongly influences the Schottky barrier at the TE-NbO_x_ interface, influencing the electronic transport mechanism and the resulting *I-V* characteristics of memristive cells in the pristine state. Asymmetric diode-like behaviours were observed in the case of Pt and Au top TE arise from the high blocking character of these metal electrodes when reversely biased. Instead, the lower pristine state resistance of devices contacted by Ir, TiN, and Nb electrodes can be ascribed to the lower blocking character of metal-oxide interface when these metals are exploited as TE. As can be observed, an almost symmetric characteristics in the case of TiN and Nb electrodes can be observed. In this context, it is worth noticing that an almost symmetric characteristic was observed in symmetrically contacted devices with Nb electrode, even if interfacial properties of the Nb BE exploited also as NbO_x_ grown substrate during anodic oxidation are in principle expected to be different from Nb TE deposited by sputtering on the previously grown NbO_x_.

**Figure 2:**
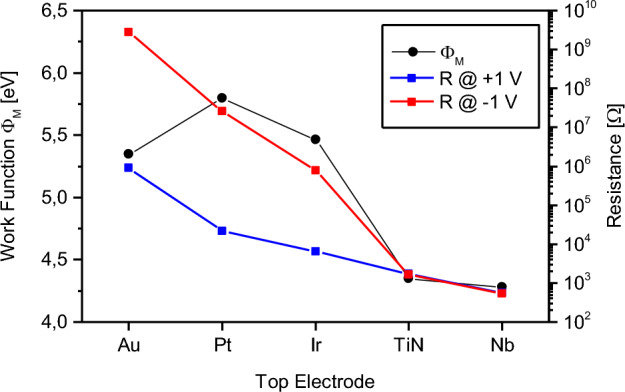
Work function Φ_M_ for the investigated TE metals (black dots), cell resistance for the different TE metal extracted at V = 1 V (blue dots) and at V = -1 V (red dots). Details on work functions can be found in Supplementary Tab. ST1.

Based on the previous discussion, the *I-V* characteristics in the pristine state can be explained on the basis of the physical properties of the hetero-junction Nb/NbO_x_/TE, in which the entity of the barriers at interfaces should reflect differences between the metal work function of the electrode and the electron affinity of the NbO_x_. In principle, the higher the barrier difference the higher the blocking character of the metal–insulator interface should result, thus limiting the electronic current in the pristine state. However, no clear trends between the pristine state resistance and the work function of the top electrode can be observed, as shown in Fig. 2 (details on the work functions and theoretical Schottky barrier height at the TE/NbO_x_ interface can be found in Supplementary Information Tab. ST1). Here, it is possible to observe that, even if Pt shows the highest theoretical barrier difference, Au-terminated devices are the ones characterised by the most insulating pristine state. Note that no clear trends in between the pristine resistance and the metal work function of electrodes were previously reported also in case of TaO_x_ switching layers^38^. A similar behaviour was also previously observed in case of Cu/CuO/TE devices where the exploitation of Ag as TE material gave rise to a more insulating pristine state with respect to Au, which on the contrary exhibits a higher work function^39^. This is because, besides the Schottky barrier height, the blocking character of the metal–insulator interface is regulated also by the interface chemistry^40^. It is worth mentioning that the interface resistance can be influenced by the presence of interfacial oxide(s)^41^. The probability for formation of an oxide can be estimated by the electronegativity (or alternatively one can referrer to the standard electrode potential) of the electrode metal, as discussed in previous works^42,43^. The resistance of the interfacial oxide is primarily determined by the point-defect structure and band structure, that determine the ionic and electronic conductivities.

### The effect of metal electrodes on the electroforming process

**Figure 3:**
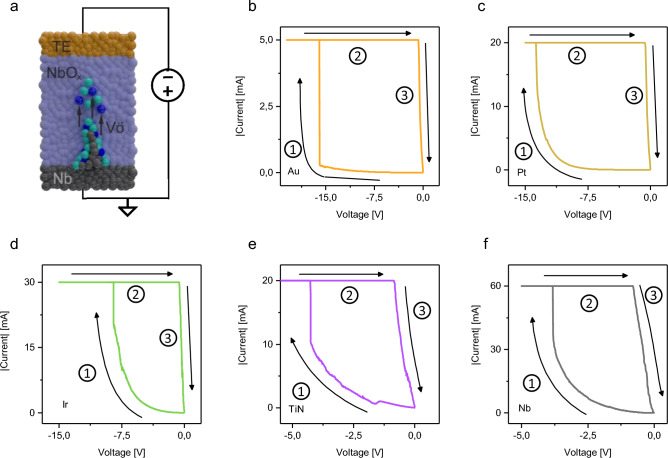
(**a**) Schematization of the forming process in a typical TE/NbO_x_/Nb VCM cell, where the process is activated by applying a negative voltage sweep on the active TE while the BE is grounded. The electroforming process rely on the formation of a sub-stoichiometric conductive channel related to the migration of ionic species inside the NbO_x_ active material (blue spheres represent the oxygen vacancies whilst the green ones indicate Nb ions in a lower valence state). Once the forming voltage is reached, the current in the *I-V* plots abruptly reaches the value of the set current compliance. Forming voltage characteristics of NbO_x_-based devices contacted by (**b**) Au, (**c**) Pt, (**d**) Ir, (**e**) TiN, and (**f**) Nb TEs. Arrows and numbers in the *I-V* curves specify the temporal evolution of the *I-V* hysteretic loop.

As a first programming step, the electroforming process is necessary to initialise the NbO_x_-based memristive cells. A schematization of the electroforming process can be found in Fig. 3a. The TE is negatively biased and subjected to a voltage sweep, in the meanwhile the Nb BE is grounded. In this case, the electroforming process can be attributed to the ionic conduction of the mobile chemical species which in this case includes both niobium and oxygen ions (or, alternatively speaking, by the oxygen vacancies) driven under the action of the electric field. Since the transport number of niobium ions is lower than the one of the oxygen ions, we can assume in principle that the process could be attributed mainly to the migration of oxygen ions^20^. In the meanwhile, the Nb in the oxide layer, reduces to its low oxidation state and a channel composed of a sub-stoichiometric NbO_x_ is expected to grow from the BE toward the TE. At the end of the process, the channel bridges the two electrodes, and the device reaches what is called the low resistance state (LRS). During this process, the oxide layer experiences a soft breakdown which permanently alters its structure. To prevent a possible hard breakdown of the device related to Joule overheating, a compliance current (CC) is externally applied to limit the maximum current allowed during the electroforming process. It is worth noticing that this mechanism can be influenced by reactions at the metal/NbO_x_ interface, as expected in case of TiN and Ir electrodes. In this context, it has been previously shown that TiN can react with transition metal oxides such as HfO to form TiOx and TiON, and similar processes may happen with NbO_x_^44^. Similarly it has been shown that Nb and Ir can interact forming Ir3Nb^45^, so the formation of compounds at the NbO_x_/Ir interface cannot be excluded.

Typical electroforming characteristics of NbO_x_ memristive cells with different TEs are reported in Fig. 3b–f. As it can be observed, for each TE metal it was possible to find proper stimulation conditions to form the cell under test. Indeed, for each TE the electroforming was characterised by an initial step in which the current follows the typical Schottky behaviour due to the barrier at the TE contact, a subsequent current jump in correspondence of the electroforming voltage where the current saturates reaching the compliance value, and a following low resistance state *I–V* characteristic going back to the origin due to the formation of the conductive channel. While a nearly linear *I–V* characteristic was observed after electroforming in Au, Pt and Ir contacted devices, the LRS state characteristics are affected by non-linearity effects in TiN and Nb contacted devices.

**Figure 4:**
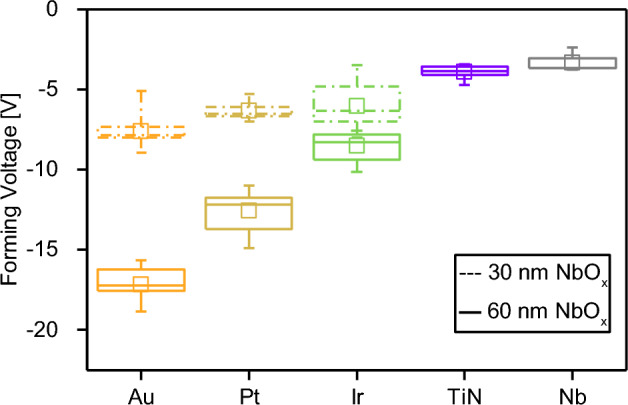
Statistical analysis of electroforming voltages of NbO_x_-based cells contacted by different TE metals. Measurements have been performed by considering the 60 nm NbO_x_ devices, with TE size of 50 × 50 µm, while dashed data refer to electroforming voltages for the 30 nm NbO_x_ devices. In boxplots, midlines represent median values, squares represent the mean values, boxes the 25th and 75th percentiles, and whiskers the minimum and maximum values.

Based on the previous results, electroforming curves of different devices have been acquired to statistically evaluate how the different TE metal affects the electroforming process. For this purpose, the forming voltage was identified as the first voltage value at which the current equals the CC value. Results of the statistical analysis are reported in Fig. 4. What is evident in this case is that TEs responsible for higher pristine state resistance result in higher forming voltages. In addition, in the case of Au, Pt, and Ir (electrodes with higher pristine state resistance), statistical analysis was performed also by considering NbO_x_ layers with a reduced 30 nm thickness, showing that a reduction of the oxide thickness results in a reduction of the electroforming voltage (details of forming curves of devices with reduced thickness in Supplementary Fig. S2). These observations are in accordance with previous works^46^, where fixed the device area, the electroforming voltage reduces by reducing oxide layer thickness, independently on the method chosen to grow the oxide layer.

### The effect of metal electrodes on resistive switching

**Figure 5:**
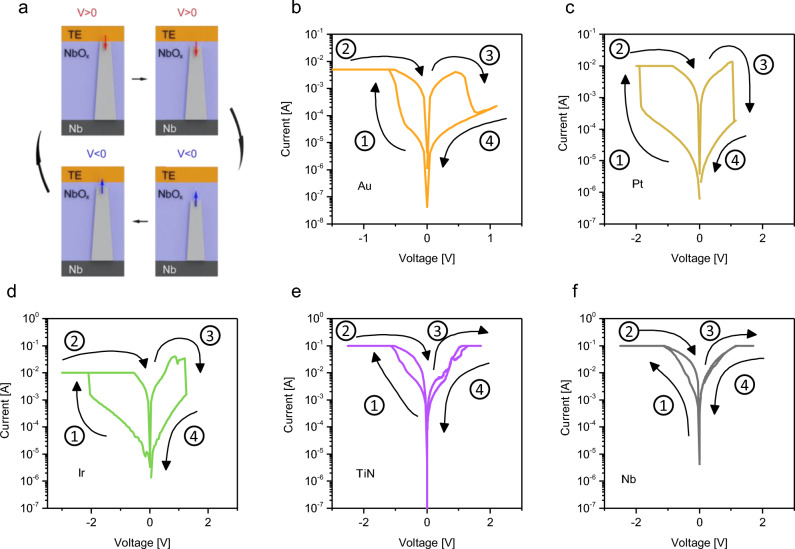
(**a**) Schematics of the switching mechanism of a VCM device by looking at the formation and rupture of the conductive channel under the application of an external bias on the top electrode (the bottom is grounded during this operation). Typical shapes of *I-V* curves for different top electrodes: (**b**) Au, (**c**) Pt, (**d**) Ir, (**e**) TiN and (**f**) Nb. The arrows and the numbers indicate the sweep direction.

Differently from the electroforming process, which is characterised by localised redox reactions that progressively lead to the formation of the conductive channel, the switching mechanism is a process that involves the localised formation/rupture of the filament previously formed, making it possible to program the devices to switch between a LRS and a high resistance state (HRS). A schematization of the switching mechanism can be found in Fig. 5a, in which a pre-electroformed device experiences the rupture of the channel near the TE electrode when a positive voltage sweep is applied, making the device switching from the LRS to the HRS (RESET process) and conversely, its restoration, with the consequent passage from HRS to LRS (SET process) when the opposite polarity voltage sweep is applied. The switching mechanism can be triggered multiple times allowing the device to experience the SET and the RESET phases in a cyclic way, resulting in the typical hysteretic characteristic of memristive devices. Figure 5b–f show typical *I–V* curves of the NbO_x_ cells contacted with different TE metals. Au and Pt terminated cells show the typical hysterical *I–V* curves which are representative of bipolar RS devices. While Ir also exhibited the capability to switching in bipolar way, these cells exhibited high instability. Instead, TiN and Nb devices, although showing some changes in the internal resistance state of the device when stimulated, did not exhibit reproducible switching characteristics after electroforming due to the permanence of the device in the ON state without the possibility of recovering the HRS. Additional data on resistive switching exemplary characteristics of unstable Ir, TiN and Nb-terminated devices can be found in Supplementary Figure S3. In this context, results suggest that for obtaining good switching capability it is necessary to have asymmetric MIM structures characterised by an ohmic contact and a low oxygen affinity metal at the counter electrode, which is in principle true for TiN, being a low oxygen affinity metal, but in the end, this one and Nb act like ohmic contact at the TE^33,47^.

### Switching behaviour of Pt and Au terminated cells

**Figure 6:**
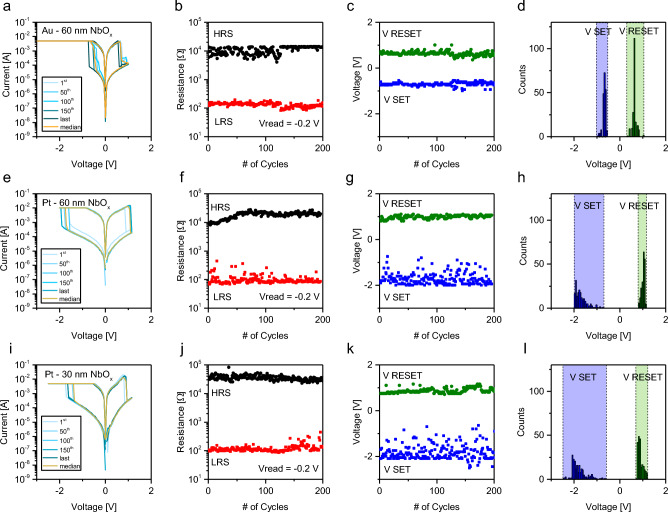
Endurance test of the good switching TE cells. First row displays results for Au TE and 60 nm NbO_x_: (**a**) some *I-V curves* of 200 cycles endurance test (cyan curves) with superimposed median (yellow curve), (**b**)HRS and LRS extracted by reading the resistance a Vread = -0.2 V, (**c**)VSET and VRESET for each cycle and (**d**) grouped in histograms. Same results reported for Pt TE and 60 nm oxide (**e**), (**f**), (**g**) and (**h**) and for Pt TE and 30 nm oxide (**i**), (**j**), (**k**) and (**l**).

Even if the switching capability was observed in each NbO_x_ cell, only the Au and the Pt terminated devices show stable switching behaviour, while the RS characteristic was observed to be unstable and characterised by low reproducibility in the case of Ir. For the Au and the Pt terminated devices we performed an endurance test, which represents one of the common figures of merit when a RS device is studied^48^. In this case, we compare the switching behaviour of the two types of metal contact tested for a 200 full-sweep cycles (measurements stopped even if the devices were able to switch in a stable way). In Fig. 6 the results of the endurance tests for devices with Au and Pt TEs. RS characteristics, endurance, SET/RESET voltages over cycling, and SET/RESET distributions in case of Nb/NbO_x_(60 nm)/Au, Nb/NbO_x_(60 nm)/Pt, and Nb/NbO_x_(30 nm)/Pt are reported in Fig. 6a–l, respectively. For the sake of completeness, results for Nb/NbO_x_(30 nm)/Au devices are reported in Supplementary Fig. S4, For all devices, *I–V* curves acquired during the test at the 1st, 50th, 100th, 150th, and at last cycle superimposed to the median on the whole test are reported.

In all cases, the RS behaviour was characterised by low cycle-to-cylce variability in terms of device LRS/HRS resistance states and SET/RESET voltages, except for Nb/NbO_x_(30 nm)/Au devices. Concerning operating voltages, Au-terminated devices are characterized by lowest and more stable SET and RESET voltages. Furthermore, a slightly higher ON/OFF ratio can be observed in case of Pt-terminated devices.

**Figure 7:**
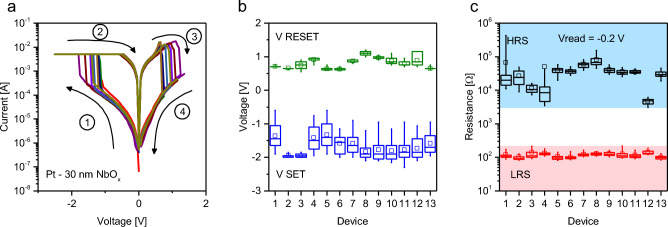
Device-to-device variability. (**a**) Representative resistive switching characteristics of different cells based on 30 nm oxide thickness devices and terminated with Pt. Curves represent the median *I-V* curve obtained over 50 cycles. Arrows and number indicate the sweep direction. (**b**) box plot collecting the SET and the RESET voltages for each endurance test. (**c**) statistical distribution of HRS and LRS collected during the endurance test for each device. Data acquired on 13 devices. Box plots were obtained from 50 consecutive cycles on the same device. Midlines represent median values, squares represent mean values, boxes the 25th and 75th percentiles, and whiskers the 5th and 95th percentiles.

While device-to-device variability of Au-terminated devices was already analysed in our previous work^25^, we have here investigated the device-to-device variability of Pt terminated devices focusing on cells with 30 nm NbO_x_. Figure 7a shows a collection of *I-V* curves representing the medians of the endurance tests for each device under test. For each device the statistical distribution of the SET and RESET voltages have been collected in Fig. 7b, showing that the RESET process endows a lower inter device and device-to-device variability compared to the SET process. Figure 7c shows the box plot distributions for the HRS and LRS acquired during each endurance test, showing that the LRS is characterised by a lower device-to-device variability compared to the HRS. For completeness, a retention test on both LRS and HRS of a 30 nm thick Pt-terminated cell is reported in Supplementary Fig. S5, showing the capability of Pt terminated devices to retain HRS and LRS for 10^3^ s.

## Conclusions

In this work, we have systematically analysed the effect of electrode metals on the switching properties of NbO_x_-based memristive cells. For this purpose, Au, Pt, Ir, TiN, and Nb electrodes have been analysed in memristive cells based on NbO_x_ grown by anodic oxidation on a Nb substrate exploited as a counter electrode. Results show that the choice of the TE is crucial for regulating the electronic transport mechanism in the pristine state as well as in regulating device electroforming and resistive switching performances. In this context, it is shown that Au and Pt represent the best choice for realising memristive devices based on NbO_x,_. It is worth noticing that further investigation, such as with temperature-dependent characterization, is required to better understand the mechanism of electronic transport regulated by interfaces. In addition, further investigation is required to understand the correlation between the thickness-dependent resistive switching characteristics and metal electrode properties.

## Experimental

### Sample fabrication

The devices have been fabricated starting from commercially available Si substrates covered by 500 nm thermal SiO_2_ (SiMat). Prior to the Nb deposition, each substrate has been sequentially washed in acetone for 4 min and in ethanol for 4 min with the assistance of an ultrasonic bath (CEIA CP102, ultrasonic cleaner). The Nb thin film deposition was realised in a DC sputtering equipped with a Nb target. The chamber was evacuated at a base pressure of 10^–7^ mbar and the thin film deposition was carried out for 5:45 min at a pressure of 3·10^–3^ mbar with the assistance of an Ar plasma to achieve a final thickness of 250 nm. To improve the adhesion of the Nb thin films, each substrate was surface-cleaned with an Ar plasma at 3·10^–2^ mbar, 25 W and 700 V for 2 min before the Nb deposition. The oxidation of the Nb thin films was achieved through anodic oxidation in a custom-made anodization cell exploiting a supersaturated solution of ammonium pentaborate in ethylene glycol prepared by mixing 76 mL of ethylene glycol (Sigma Aldrich), 13 g of ammonium pentaborate (Sigma Aldrich) and 100 mL of deionized water. The solution was stirred overnight and filtered two times to completely remove the unreacted reagents. The solution was stirred before each anodization process. Only the central area of each sample was oxidized in a circular shape of radius 0.6 mm, corresponding to the exposed area to the electrolytic solution. The anodization process was carried out by applying a constant current of 1 mA provided by a Keithley 220 current source meter until the anodizing voltage was reached, then the current was adjusted to keep the voltage constant to the anodizing voltage. The anodizing process lasted 300 s from the voltage plateau. During the whole anodization process, in order to establish contact with the back side of the sample, a Cu-conductive tape was used and a Labview interface automatically adjusted the current in the meanwhile the voltage was reached at the plateau (voltage read through HP 34401a). Two different anodizing voltages were chosen, 20 V and 10 V, corresponding to the final NbO_x_ thicknesses of 60 nm and 30 nm respectively. The thickness of the so-grown NbO_x_ films was evaluated by means of spectroscopic ellipsometry (alpha-SE Ellipsometer J.A. Woollam). The final 50 × 50 µm^2^ TEs were defined by optical lithography and the deposition of the different TE materials was realised through DC sputtering (details on the sputtering deposition can be found in Supplementary Information Tab. ST2).

### Electrical measurements

All the samples were measured following the same contact scheme: Nb bottom electrode grounded, and voltage directly applied on the top electrode. For the electrical characterization, a probe station equipped with two tungsten tips was used. Electrical measurements were acquired both using a Keithley 4200A-SCS parameter analyser and Keithley 6430. Pristine state curves were acquired by applying a voltage sweep in the range [-1,1] V, starting from the value − 1 V toward 1 V and coming back to the initial value, assuming a voltage step of 0.01 V. For the statistical analysis of electroforming voltages, 12 Au-terminated cells with NbO_x_ 60 nm, 10 Au-terminated cells with NbO_x_ 30 nm, 10 Pt-terminated cells with NbO_x_ 60 nm, 15 Pt-terminated cells with NbO_x_ 30 nm, 15 Ir-terminated cells with NbO_x_ 60 nm, 10 Ir-terminated cells with NbO_x_ 30 nm, 12 TiN-terminated cells with NbO_x_ 60 nm, and 12 Nb-terminated cells with NbO_x_ 60 nm. For the evaluation of the forming voltage acquired the first voltage value at which the current became equal to the CC chosen for that specific TE device. The HRS and the LRS resistances were directly extracted by the ratio V/I assuming a V read = − 0.2 V, during each cycle for all the endurance tests. The SET voltages were extracted by reading, for each cycle, the first voltage at which the current equals the CC in the negative polarity, whilst the RESET one is assumed as the voltage at which the current reaches the maximum positive value.

### Supplementary Information

Supplementary Information.

## Data Availability

The data that support the findings of this study are available on Zenodo (https://doi.org/10.5281/zenodo.8288341). All other data are available from the authors.
