# Supplementary material for: Effect of electrode materials on resistive switching behaviour of NbOx-based memristive devices
Source: Sci Rep. 2023 Oct 9;13:17003. doi: 10.1038/s41598-023-44110-w (PMC10562416; doi:10.1038/s41598-023-44110-w)
Supplement: Supplementary file 1 — Supplementary Information. [file 41598_2023_44110_MOESM1_ESM.docx]

**Effect of electrode materials on resistive switching behaviour of NbO_x_-based memristive devices – Supplementary information**

Giuseppe Leonetti^1^, Matteo Fretto^2^, Fabrizio Candido Pirri^1^, Natascia De Leo^2^, Ilia Valov^3,4,*^, and Gianluca Milano^2,*^

^1^ Politecnico di Torino, Department of Applied Science and Technology (DISAT), C.so Duca degli Abruzzi 24, 10129, Turin, Italy.

^2^ Istituto Nazionale di Ricerca Metrologica (INRiM), Advanced Materials Metrology and Life Sciences Division, Strada delle cacce 91, 10135 Turin, Italy

^3^ Forschungszentrum Jülich, Institute of Electrochemistry and Energy System, WilhelmJohnen-Straße, 52428, Jülich, Germany.

^4^ “Acad. Evgeni Budevski” (IEE-BAS, Bulgarian Academy of Sciences (BAS), Acad. G. Bonchev Str., Block 10, 1113 Sofia, Bulgaria

Emails: [i.valov@fz-juelich.de](mailto:i.valov@fz-juelich.de), [g.milano@inrim.it](mailto:g.milano@inrim.it)

**Table ST1.** Comparison of metal work functions and theoretical Schottky barrier at the TE/NbOx interface.

| TE | Work Function Φ_M_ | Barrier Φ_B_ = Φ_M_-χ | Type of contact at TE interface | References |
| --- | --- | --- | --- | --- |
| Ir | 5.25 eV | 1.45 eV | Schottky | [1] |
| Au | (5.1÷5.4) eV | 1.45 eV | Schottky | [1],[2] |
| Pt | (5.7÷6.35) eV | 2.23 eV | Schottky | [1],[2] |
| TiN | (4.2÷4.5) eV | 0.55 eV | Ohmic | [3] |
| Nb | 4.3 eV | 0.5 eV | Ohmic | [2] |

**Table ST2**. The deposition on the Nb BE and the anodization process was carried out at INRiM laboratories. Nb and Au TE were deposited at INRiM laboratories, whilst, TiN ,Pt and Ir were deposited at RWTH.

| TE material | Nominal Ox. Thickness | target | P_dep_ | Power | Flux Ar | Flux N_2_ | Nominal Thickness |
| --- | --- | --- | --- | --- | --- | --- | --- |
| TiN | 60 nm | Ti | 1·10^-3^mbar | 200 W | 27 sccm | 3 sccm | 50 nm |
| Ir | 60 nm | Ir | 2·10^-3^mbar | 30 W | 20 sccm | - | 50 nm |
| Pt | 60 nm | Pt | 5·10^-3^mbar | 200 W | 30 sccm | - | 50 nm |
| Ir | 30 nm | Ir | 2·10^-3^mbar | 30 W | 20 sccm | - | 50 nm |
| Pt | 30 nm | Pt | 5·10^-3^mbar | 200 W | 30 sccm | - | 50 nm |
| Au | 60 nm | Au | 5·10^-3^mbar | 100 W | 53 sccm | - | 150 nm |
| Au | 30 nm | Au | 5·10^-3^mbar | 100 W | 53 sccm | - | 150 nm |
| Nb | 60 nm | Nb | 3·10^-3^mbar | 110 W | 50 sccm | - | 150 nm |

**Supplementary Figure S1**

**Figure S1**. Log scale curve for the pristine state reported in Fig.1 b-f. Curves refer to 60 nm thick NbO_x_ devices.

**Supplementary Figure S2**

**Figure S2**. Example of the electroforming curves for the 30 nm oxide devices for the high work function AE. In the order (**a**) Ir, (**b**) Au and (**c**) Pt. Arrows and numbers indicate the direction of the process.

**Supplementary Figure S3**

d

a

c

b

**Figure S3**. (**a**) Example of instable switching behaviour of an Ir- terminated cell: after few voltage sweeps, either in the positive or in the negative polarity, the device is not able to correctly SET in a LRS or RESET in HRS. (**b**) Another example of I-V characteristics of Ir-terminated devices showing the impossibility to recover the HRS after electroforming process. A similar unstable behaviour was observed in case of TiN (**c**) and Nb (**d**) terminated devices. Numbers in the figures describe the evolution of the I-V characteristic.

**Supplementary Figure S4**

**Figure S4**. Resume of the endurance test of 30 nm oxide devices terminated with Au top electrode. (**a**) exemplary curve of a typical I-V cycle. (Arrows and numbers indicate the direction of the voltage sweep) (**b**) HRS and LRS recorded over an endurance test showing the failure of this devices into face high endurances, in this case the device was not able to SET anymore after about 70 cycles. boxplot of SET and RESET voltages (**c**) and HRS and LRS (**d**) of some devices able to switch. For a comparative analysis only devices with at least 20 cycles endurance were considered. Read voltage is -0.2 V for the HRS and LRS boxplot.

**Supplementary Figure S5**

**Figure S5**. An example of a retention test for a 30 nm NbO_x_ device terminated with Pt. The test was carried out for 1000 s.

**References**

[1] B. Ofuonye, J. Lee, M. Yan, C. Sun, J. M. Zuo, and I. Adesida, “Electrical and microstructural properties of thermally annealed Ni/Au and Ni/Pt/Au Schottky contacts on AlGaN/GaN heterostructures,” *Semicond Sci Technol*, vol. 29, no. 9, Sep. 2014, doi: 10.1088/0268-1242/29/9/095005.

[2] W. M. Haynes. CRC Handbook of Chemistry and Physics. (CRC press). p. 12-124 (95^th^ ed.)

[3] L. P. B. Lima, J. A. Diniz, I. Doi, and J. Godoy Fo, “Titanium nitride as electrode for MOS technology and Schottky diode: Alternative extraction method of titanium nitride work function,” in *Microelectronic Engineering*, Apr. 2012, pp. 86–90. doi: 10.1016/j.mee.2011.04.059.
